# Association between nutritional status and the immune response in HIV + patients under HAART: protocol for a systematic review

**DOI:** 10.1186/2046-4053-3-9

**Published:** 2014-02-10

**Authors:** Maryline Sicotte, Étienne V Langlois, Joséphine Aho, Daniela Ziegler, Maria Victoria Zunzunegui

**Affiliations:** 1 Research Center of the Montreal University Hospital Center (CRCHUM), 850 rue St-Denis, tour St-Antoine, Montreal, QC H2X 0A9, Canada; 2 School of Public Health, Montreal University, Pavillon 7101 Parc Avenue, Montreal, QC H3C 3 J7, Canada; 3 Institute of Tropical Medicine, Nationalestraat 155-2000, Antwerp, Belgium; 4 Documentation, Montreal University Hospital Center (CHUM), Montreal, QC Canada; 5 Montreal University Public Health Research Institute (IRSPUM), Montreal, QC Canada; 6 Documentation Center, Montreal University Hospital Centre (CHUM), Saint-Luc Hospital, 1058 rue Saint-Denis, Principal Pavilion, 1st floor #1303, Montreal, QC H2X 3 J4, Canada

**Keywords:** Malnutrition, CD4, HIV, Highly active antiretroviral therapy, Low- and middle-income countries, Systematic review, Protocol

## Abstract

**Background:**

Over 850 million people worldwide and 200 million adults in Sub-Saharan Africa suffer from malnutrition. Countries most affected by HIV are also stricken by elevated rates of food insecurity and malnutrition. HIV infection and insufficient nutritional intake are part of a vicious cycle that contributes to immunodeficiency and negative health outcomes. However, the effect of the overlap between HIV infection and undernutrition on the immune response following antiretroviral initiation remains unclear. A possible explanation could be the lack of consensus concerning the definition and assessment of nutritional status. Our objectives are to investigate the existence of an association between undernutrition and immune response at antiretroviral treatment initiation and the following year in low- and middle-income countries where malnutrition is most prevalent.

**Methods/design:**

Our systematic review will identify studies originating from low- and middle-income countries (LMICs) published from 1996 onwards, through searches in MEDLINE (PubMed interface), EMBASE (OVID interface), Cochrane Central (OVID interface) and grey literature. No language restrictions will be applied. We will seek out studies of any design investigating the association between the nutritional status (for example, undernourished versus well nourished) and the immune response, either in terms of CD4 count or immune failure, in seropositive patients initiating antiretroviral therapy or in their first year of treatment. Two reviewers will independently screen articles, extract data and assess scientific quality using standardized forms and published quality assessment tools tailored for each study design. Where feasible, pooled measures of association will be obtained through meta-analyses. Results will be reported according to the Preferred Reporting Items for Systematic Reviews and Meta-Analyses (PRISMA) Statement. This protocol has been registered in the PROSPERO database (registration number: CRD42014005961).

**Conclusion:**

Undernutrition and weight loss are prevalent amongst highly active antiretroviral therapy (HAART)-treated patients in LMICs and contribute to excess early mortality. A possible intermediate pathway could be poor immune reconstitution secondary to deficient nutritional status. In the face of limited access to second line treatments, raising HIV resistance and cut backs to HIV programs, it is crucial to identify the factors associated with suboptimal response and therapeutic failure in order to better customize the care strategies employed in LMICs.

## Background

Malnutrition is a persisting public health issue affecting over 850 million people worldwide and 200 million adults in Sub-Saharan Africa (SSA) [1,2]. The term 'malnutrition’ is often used indifferently to refer both to undernutrition and overnutrition. In undernutrition, macro- and/or micronutrient supply are continuously below minimum dietary requirements, which leads to changes in body composition and diminished function [3,4]. For the purpose of this systematic review, we will focus on undernutrition as a state of malnutrition.

Before the HIV epidemics, malnutrition was the first cause of immunosuppression worldwide [5]. Micro- and macronutrient deficiencies weaken the immune system through atrophy of the thymus and lymphoid tissue, alteration of T cell subsets, decrease of T cell counts and cytokine response [6,7]. In protein energy malnutrition (PEM), cell-mediated and innate immunity [6,8] are particularly lessened, thereby increasing the risk of viral infection (including HIV) and disease progression [9-11]. Furthermore, persistent malnutrition leads to an inflammatory state through decreased leptin levels [12] and altered intestinal mucosa permeability which further augments the organism’s vulnerability to infection [5].

Countries where food insecurity and malnutrition are common are also stricken by high HIV incidence and prevalence rates [13,14]. The effect of overlap between HIV infection and undernutrition in low- and middle-income countries (LMICs) on immunological function remains unclear [2,14,15]. HIV is a cause of nutritional deficiencies through its effect on nutrient absorption - for example, HIV-mediated inflammation and induced mucosal immune cell damage in the gastrointestinal tract leads to diarrhea and malabsorption, decreased intake - for example, odynophagia induced by esophageal candidiasis (an opportunistic infection (OI)) - limits the capacity to feed oneself. Finally, HIV itself or associated OI can result in increased energy expenditure [16-18]. As such, malnutrition and HIV coexist in a vicious perpetuating cycle.

Increased availability of highly active antiretroviral therapy (HAART) in LMICs has led to some improvement of the nutritional status of patients [19-22]. However, for certain individuals, undernutrition and weight loss persist despite therapy [17,23,24]. Just like with HIV, HAART and malnutrition contribute to a deadly cycle. Highly active antiretroviral therapy (HAART) leads to increased requirements for macro- and micronutrients, high metabolic demands [25] and low appetite [26] which perpetuate undernutrition [27]. Simultaneously, malnutrition exacerbates side effects [28-31], alters drug pharmacokinetics [32], and impinges on adherence [33] thereby limiting the beneficial effects of the therapy. Like in ART-naive patients, suboptimal nutritional status amongst those treated is associated with disease progression and early death [34-41]. As such, excess early mortality in patients initiating treatment in LMICs compared to high-income countries has been reported [42].

Despite theoretical and experimental evidence linking malnutrition and altered immune function, there exists no consensus concerning the impact of malnutrition on the immune response in HIV patients receiving tri-therapy. Numerous nutritional interventions have been attempted in seropositive populations under antiretroviral treatment (ART) without convincing clinical results. As such, a recent systematic review investigating nutritional interventions and clinical outcomes in high-, middle- and low-income countries failed to show any advantages in terms of CD4 or weight gain in those receiving food supplements [13]. In populations from LMICs, research has failed to demonstrate an association between lower CD4 counts and markers of undernutrition amongst newly diagnosed seropositive individuals compared to seronegative controls [43,44]. In populations under combined antiretroviral treatment (cART), evidence so far appears contradictory. As underlined by Soeters (2008), a possible explanation for this absence of consensus could be the lack of homogeneity in the definition and conceptualization of malnutrition [4]. Various undernutrition syndromes have been grouped under the umbrella of malnutrition - protein-energy malnutrition (PEM), cachexia, sarcopenia, marasmus, kwashiorkor - each being the result of different pathophysiological processes and each leading to distinct morphological and biochemical modifications [4,45]. Furthermore, there exists no unique indicator to capture malnutrition. As such, prevalence of malnutrition amongst HIV patients varies according to the instrument used [46]. In field studies, body composition is often evaluated using either body mass index (BMI), mid-upper arm circumference (MUAC), skinfolds (SK), or bioelectrical impedance (BIA); each method capturing a different aspect of body composition. The simplicity and cheapness of anthropometric techniques has made them popular in nutritional studies, independently of their appropriateness [47]. Some authors indicate that nutritional assessment should also include analysis of biochemical nutritional parameters such as albumin and C-reactive protein (CRP) to account for inflammation in the establishment and maintenance of malnutrition [4,48].

Further evidence is required to clarify the association between nutritional status and immunological outcomes. For the purpose of this systematic review, we will use the operationalized definition of malnutrition proposed by Soeters (2008). In his model, Soeters attempts to regroup different pathophysiological explanations and consequences of malnutrition into one conceptual framework. This model feeds onto the different definitions that have been proposed to describe malnutrition. We will focus on the clinical assessment of body composition and inflammatory activity [4]. For the former, indicators investigated will include BMI, MUAC, SK, and BIA (as a measure of body cell mass (BCM)). As for the latter, markers investigated will include albumin, hemoglobin and C-reactive protein (CRP). These measures will be contrasted with one measure of function, namely the immune function. With this systematic review we hope to provide clarification concerning the cross-sectional and longitudinal associations between undernutrition and immune response in seropositive patients receiving ART in LMICs.

### Objectives and research questions

This systematic review is guided by the following research hypotheses: a) macro-nutritional deficiencies in HIV + individuals, either at time of treatment initiation or in the first year of treatment, will lead to a defective immune response to HAART independently of disease stage, and b) the existence and strength of the associations observed between malnutrition and immune reconstitution following ART initiation will vary with the choice of nutritional marker.

Our aim is to investigate the existence of an association between undernutrition and the immune response following ART initiation in LMICs. To do so, we will identify, assess and synthesize the literature from LMICs a) to evaluate the cross-sectional relationship between malnutrition and immune markers at treatment initiation; b) to investigate the association between nutritional indicators at time of treatment initiation and the prospective immune response in the year after ART initiation and, c) to assess longitudinal co-variation between nutritional and immune trajectories in patients under HAART for less than a year; and d) to establish whether the associations highlighted in a), b), and c) vary according to individual and/or subgroups of nutritional indicators.

## Methods

**Figure 1:**
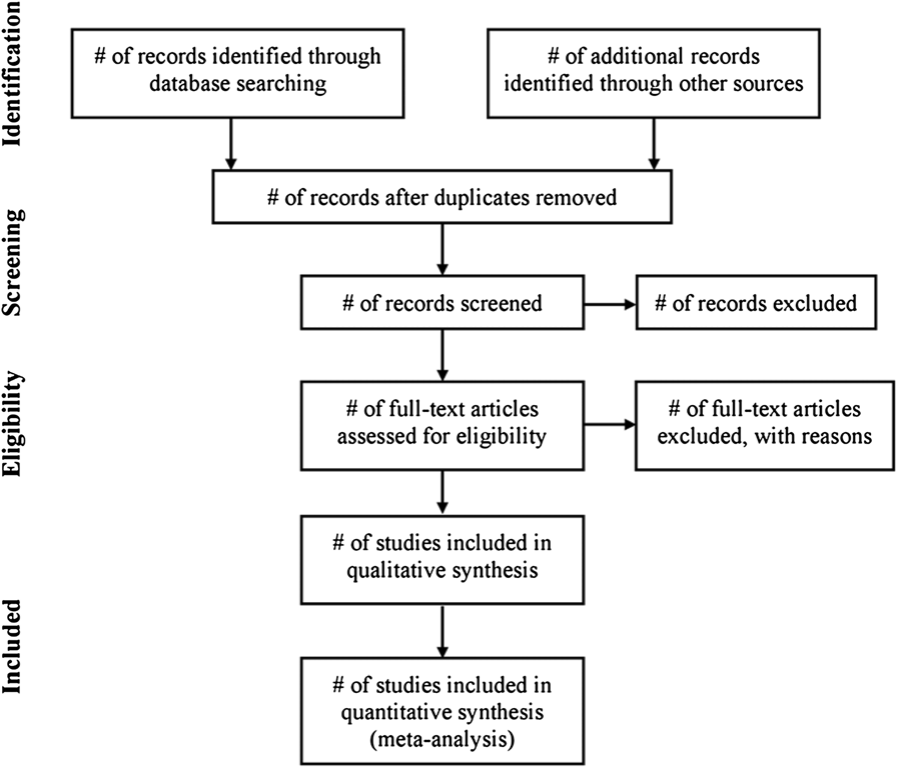
**PRISMA flow diagram.** Source*:* Moher, D., *et al*., *Preferred reporting items for systematic reviews and meta-analyses: the PRISMA statement.* BMJ, 2009. **339**: p.b2535.

The guidelines proposed in the Preferred Reporting Items for Systematic Reviews and Meta-Analyses (PRISMA) statement will be used as a template for the development and execution of this review (Figure 1) [49]. The methodological choices described in this protocol have been inspired by previously published work from members of our group [50]. This systematic review protocol was registered with the PROSPERO database (registration number: CRD42014005961).

### Search strategy

The search strategies will be carried out by the research team (MS, JA, and MVZ) and an expert librarian (DZ). No language restriction will be enforced conditional to the provision of an English abstract. Using specific medical subject headings (MeSH) and text words, we will identify studies originating from LMICs published from 1996 onwards (year the use of HAART became standard [51]) through searches MEDLINE (PubMed interface), EMBASE (OVID interface), all EBM reviews from OVID (Cochrane Database of Systematic Reviews <2005 to September 2013>, EBM Reviews - ACP Journal Club <1991 to September 2013>, EBM Reviews - Database of Abstracts of Reviews of Effects <third quarter 2013>, EBM Reviews, Cochrane Central Register of Controlled Trials <September 2013>, EBM Reviews - Cochrane Methodology Register <third quarter 2012>, EBM Reviews - Health Technology Assessment <third quarter 2013>, EBM Reviews - NHS Economic Evaluation Database <third quarter 2013>), and Cochrane Central (manual search). Details concerning our exact search strategy are provided in Additional file 1. We will also screen grey literature sources, namely: Social Care Online; National Institute for Health and Clinical Excellence (NICE); System for Information on Grey Literature in Europe (OpenSigle); National Guideline Clearing House; Health Development Agency; National Institutes of Health; Research Service Delivery and Organization Program (SDO); Research Register for Social Care; Google Scholar and OpenGrey. Furthermore, we will search official Websites of institutions active in the fields of HIV/AIDS and nutrition, along with bibliographic references of retrieved articles and reviews.

Our search strategy will combine the following subheadings: 1) Nutrition/nutritional indicators; 2) Antiretroviral treatment; 3) Immune response and 4) Low- and middle-income countries. The terms 'HIV’ and 'AIDS’ will not be included in the search strategy as we consider these notions to be captured under the second theme. All articles and reports retrieved during the identification phase will be combined in an Endnote file. Duplicates will then be extracted. Endnote entries will be further filtered to remove all pediatric and peri/postpartum literature. Details concerning these steps can be found in Additional files 1 and Additional file 2*.*

### Study inclusion criterions

#### Participants and setting

We will identify studies investigating the immune response according to nutritional markers in non-pregnant, treatment-naive, HIV-1 infected adults Participants will either be initiating HAART - a regimen consisting of three antiretroviral molecules - or receiving HAART for less than a year. All regimen composition will be considered. Malnutrition in LMICs is a great public health concern with prevalence as high as 69% in the general population [52]. For this reason, we will focus on studies originating from these settings. We will use The World Bank Group’s classification to identify those countries (Additional file 2) [53].

#### Types of designs

We will include experimental studies, namely, randomized controlled trials (RCTs) and cluster-randomized trials (CRTs); quasi-experimental studies including quasi-randomized trials, controlled before-after studies (CBAs) and interrupted time series studies (ITSs); as well as observational studies including cohort, case-control and cross-sectional studies.

#### Study exclusion criteria

Studies assessing children or adolescent, pregnant women, individuals either co-infected or solely infected with HIV-2 will be excluded. Also, patients that are not about to start treatment or who have been on HAART for longer than a year will not be considered. Studies strictly reporting qualitative results will not be retained

#### Outcomes

Our primary outcome will be the immune response in the year following ART initiation either in terms of CD4 counts or percentage, or change in CD4 count between visits (either categorized or in continuous form). Secondary outcomes will include: 1) immune treatment failure and 2) time to immune failure after ART initiation. All definitions of immune treatment failure will be considered providing they are solely based on CD4 response.

#### Determinants

Our main exposure will be malnutrition at time of treatment initiation and in the first year of treatment. As suggested by Soeters (2008), indicators of body composition and inflammatory activity will be considered, namely: weight, BMI, MUAC, SK, and BIA (a measure of BCM, %BCM, phase angle, extracellular water (ECW), intracellular water (ICW) and fat-free mass (FFM)) for the former category; and albumin, hemoglobin and C-reactive protein (CRP) for the latter [4]. Variables other than weight should be categorized according to validated cut-off values capable of identifying malnourished individuals in the populations at hand. Interpretation of BIA parameters should be made in relation to age and BMI-adapted reference values for each given parameter. Only longitudinal variations in weight following ART initiation will be considered. Wasting syndrome will not be an item of interest because it refers to a cluster of symptoms that goes beyond simple malnutrition [17,54].

### Study selection procedure

#### Screening and eligibility

Study selection will be done in two stages. First, two independent researchers (JA (epidemiologist, PhD) and MS (PhDc)) will screen entries on titles and abstracts for relevance and presence of the selection criteria listed above. Articles will then be classified as i) included, ii) excluded or iii) uncertain. The inter-rater agreement will be computed using Cohen’s Kappa before initiating the second stage. A sample size of 226 abstracts will be required for the calculations assuming a significance level α of 0.05 (z = 1.96), a significance level β of 0.20 (z = 0.842), κ_0_ = 70%, κ_1_ = 90%, and a positive rating of 10% per rater [55]. Second, the full text articles of selected abstracts (records categorized as included or uncertain) will be independently reviewed for further eligibility analysis using a standardized form with explicit inclusion and exclusion criteria (Additional file 3). Discordance between the authors will be resolved by consensus or through discussion with a senior epidemiologist (MVZ).

#### Data collection process

We wish to retrieve quantitative results exploring the association between the nutritional status and immune response as described in our objectives. As such, an association of measure - either frequency ratio or statistical test comparing a marker of malnutrition contained in the list mentioned above and a measure of the immune response - has to be provided. Survival analyses will also be considered - for example, time to immune failure according to nutritional status at baseline. Reviewers will use a standardized data collection form to extract data such as study characteristics (country, setting, year of publication, study design, sample size); participants’ characteristics (age, sex, ART-naive or not, time since initiation of ART, type of ART regimen, other medication); outcomes (CD4 counts or percentage, change in CD4 counts, immune failure, definition of immune failure (if applicable)); and measures of the association between immune response and nutritional indicators with information concerning adjustments (univariate versus multivariate analyses, confounding variables) (Additional file 3). Initially, the form will be piloted on a random sample to examine its reliability in data abstraction. According to the results, and if necessary, the form will be refined. JA and MS will independently extract the data. Unsettled disagreements will be resolved by discussion with a senior reviewer (MVZ).

### Assessment of risk of bias and methodological quality

The scientific quality of individually selected articles will be appraised to identify studies with the best internal validity. To do so, standardized quality assessment tools tailored to each study design will be used to best assess methodological quality and risk of spurious associations. The Cochrane Collaboration’s Risk of Bias Tool (CCRBT) [56] and Cochrane Effective Practice and Organization of Practice (EPOC) Risk of Bias Tool [57] will be used for RCTs and quasi-experimental designs respectively. For cohort, case control and cross-sectional studies, we will use the Effective Public Health Practice Project (EPHPP) Quality Assessment Tool for Quantitative Studies with extended selection bias assessment. The latter encompasses the principal quality items identified by the *Strengthening the Reporting of Observational Studies in Epidemiology (STROBE) Statement*[58]. As suggested by Groenwold and Rovers (2010), adherence to the STROBE statement assures that the presentation of study characteristics is done to facilitate scientific appraisal [59]. Domains of importance will include: explicit identification of the study population, methods of outcome and determinant assessment, attrition rate and losses to follow-up, incomplete outcome data, as well as other sources of bias. Quality assessment will be carried out independently by each reviewer (JA and MS). Disagreements will be resolved by discussions between reviewers and with a senior advisor (MVZ) if necessary. Inter-rater reliability will be computed using Cohen’s Kappa to measure the interobserver agreement.

Studies investigating nutritional factors and immune response in LMIC are greatly prone to publication bias due to language barriers, limited funding, limited interest, and so on. For this reason, grey literature will be extensively scrutinized to uncover relevant data. Furthermore, authors of the retained articles will be contacted directly to insure that no negative results have been withheld from publishing. These issues will be discussed in length in the systematic review. If enough studies within any given design (cross-sectional, case-control, cohorts) are identified to conduct a meta-analysis, funnel plots and trends tests will be used to assess publication bias.

### Data synthesis

If the studies uncovered allow it, associative measures between malnutrition and immune indicators will be pooled through meta-analyses using The Cochrane Group’s Review Manager software (RevMan 5.1) [60]. Data will be combined and analyzed by markers of undernutrition (BMI, MUAC, SK, BIA, albumin, hemoglobin or CRP) and/or subgroups of indicators (body composition versus inflammation). Where possible, and as suggested by Deeks (2002) and the *Cochrane Handbook for Systematic Reviews of Interventions*, summarized measures of effectiveness will be presented as such: for dichotomized outcomes (for example, presence/absence of immune failure), odds ratios or risk ratios and their respective confidence intervals will be used. For continuous outcomes, mean difference and its associated standard error will be used. For longitudinal studies, we will present risk ratios or hazard ratios. Odds ratios will be used in cross-sectional or case-control studies. Categorical variables will be meta-analyzed in a dichotomous form [61,62].

Graphical summaries of study estimates and aggregated direct estimates will be presented for each nutritional determinant if multiple studies are uncovered. We will not perform indirect comparisons. We believe that such analyses are inappropriate in our case due to the numerous potential confounders and effects modifiers unaccounted for in the methodology and/or analyses that are likely to differ between settings and studies.

The statistical heterogeneity across studies will be investigated using the I^2^ statistic. This statistic describes the percentage of total variation between studies that is due to sampling rather than methodological error [63]. Heterogeneity will be considered statistically significant if I^2^ is over 50%. In the case of substantial heterogeneity, random, rather than fixed-model effects will be used [64]. Separate sensitivity and/or sub-analyses will be conducted to assess heterogeneity across methodological factors such as study design, overall study quality, length of follow-up, geographical region or type of variables. If possible, we will carry out separate meta-analyses of adjusted versus non-adjusted (or insufficiently adjusted) association measures. Reasons for clinical heterogeneity will be explored qualitatively *a priori* based on known literature. However, many known and unknown confounders and effect modifiers are likely to affect the association between malnutrition and immune response. For instance, it is possible that the presence of other viral or bacterial infections, detected or not, modifies the strength of the associations between malnutrition and response to HAART. Where conditions impede meta-analyses, data will be synthesized narratively. Our results will be presented according to the PRISMA Statement [49].

Finally, and if possible, quality of the overall evidence will be evaluated by aggregating quality ratings of individual studies using the GRADE working group system [65].

## Discussion

Although increased availability of first line HAART in LMICs has significantly reduced mortality and morbidity amongst people living with HIV/AIDS (PLA), undernutrition and weight loss persist [17,23,24]. Both act as strong predictors of excessive early mortality [35,37,40,41]; possibly through poor immune reconstitution secondary to deficient nutritional status in PLA. With the rapid scale-up of ART programs, concerns have also been growing about the sustainability and effectiveness of such programs [14]. Currently, funds injected into HIV programs are diminishing and access to second line regimens in LMICs continues to be restricted. There is thus a growing need to optimize therapeutic success of first line ART, delay the need for therapeutic switch, and limit rates of resistance, which are on the rise, in those regions [66].

With this systematic review we hope to clarify the association between malnutrition and immunological response to HAART in HIV + population. More specifically, we wish to establish whether ART drug treatment is suboptimal in the undernourished. Our clinical goals are to: 1) facilitate the identification of patients at greater risk of inadequate immune response; and 2) help in differentiating between malnutrition in PLA and more advanced disease (that is greater inflammatory process). We believe our results will contribute to informing physicians and policy makers in settings where care strategies have to be optimized to limit therapeutic failure by shedding some light on the processes that contribute to suboptimal therapeutic response. We are aware that the studies we will uncover will present different limitations due to the complexity of the association of interest and the challenges associated with nutritional research in vulnerable groups. These limitations, as well as bias and precision of estimates, will be discussed in length in our review giving special attention to the possible publication bias of studies conducted in settings with limited resources and in non-English speaking populations by non-English speaking researchers. As suggested by the PRISMA statement, we wish this protocol to be part of an iterative process that respects accepted and transparent reporting methods [49].

## Abbreviations

ART: antiretroviral; BCM: body cell mass; BIA: bioelectrical impedance; BMI: body mass index; cART: combined antiretroviral treatment; CBAs: controlled before-after studies; CCRBT: Cochrane Collaboration’s Risk of Bias Tool; CRP: C-reactive protein; CRTs: cluster-randomized trials; ECW: extracellular water; EPHPP: Effective Public Health Practice Project; EPOC: Cochrane Effective Practice and Organization of Practice; FFM: fat-free mass; HAART: highly active antiretroviral; HIV: human immunodeficiency virus; ICW: intracellular water; ITSs: interrupted time series studies; LMIC: low- and middle-income countries; MUAC: mid-upper arm circumference; NICE: National Institute for Health and Clinical Excellence; OI: opportunistic infection; PEM: protein energy malnutrition; PLA: people living with HIV/AIDS; PRISMA: Preferred Reporting Items for Systematic Reviews and Meta-Analyses; RCT: randomized controlled trial; SK: skinfold; SSA: Sub-Saharan Africa; STROBE: Strengthening the Reporting of Observational Studies in Epidemiology.

## Competing interests

The authors have no financial or non-financial competing interests.

## Authors’ contribution

MS, EVL, JA and MVZ contributed to the conception and design of the review. MS and DZ developed the search strategies. MS and EVL drafted the manuscript. JA, EVL and MVZ were actively involved in critically revising the protocol for important intellectual content. DZ made a substantial contribution to the *Information sources and literature search* section, and to Additional file 1. All authors read and approved the final protocol.

## Author’s information

MS is a MD/PhD candidate currently completing her dissertation on the subject of nutrition in HIV research at Montreal University.

## Supplementary Material

Additional file 1 Search strategy MEDLINE, EMBASE, and Cochrane Central. Click here for file

Additional file 2 Endnote file manipulations. Click here for file

Additional file S Data collection form. Click here for file
