# Supplementary material for: Association between nutritional status and the immune response in HIV + patients under HAART: protocol for a systematic review
Source: Syst Rev. 2014 Feb 10;3:9. doi: 10.1186/2046-4053-3-9 (PMC3922999; doi:10.1186/2046-4053-3-9)
Supplement: Additional file 1 — Search strategy MEDLINE, EMBASE, and Cochrane Central. [file 2046-4053-3-9-S1.pdf]

Annexe 1. Search Strategies

1. MEDLINE (PubMed interface, 1980 onwards)

(((("developing countries"[MeSH Terms] OR ("developing"[All Fields] AND "countries"[All Fields]) OR "developing countries"[All Fields]) OR ("developing countries"[MeSH Terms] OR ("developing"[All Fields] AND "countries"[All Fields]) OR "developing countries"[All Fields] OR ("developing"[All Fields] AND "country"[All Fields]) OR "developing country"[All Fields]) OR ("developing countries"[MeSH Terms] OR ("developing"[All Fields] AND "countries"[All Fields]) OR "developing countries"[All Fields] OR ("underdeveloped"[All Fields] AND "countries"[All Fields]) OR "underdeveloped countries"[All Fields]) OR ("developing countries"[MeSH Terms] OR ("developing"[All Fields] AND "countries"[All Fields]) OR "developing countries"[All Fields] OR ("underdeveloped"[All Fields] AND "country"[All Fields]) OR "underdeveloped country"[All Fields]) OR (emergent[All Fields] AND countries[All Fields]) OR (emergent[All Fields] AND country[All Fields]) OR ("developing countries"[MeSH Terms] OR ("developing"[All Fields] AND "countries"[All Fields]) OR "developing countries"[All Fields] OR ("developing"[All Fields] AND "nation"[All Fields]) OR "developing nation"[All Fields]) OR ("developing countries"[MeSH Terms] OR ("developing"[All Fields] AND "countries"[All Fields]) OR "developing countries"[All Fields] OR ("developing"[All Fields] AND "nations"[All Fields]) OR "developing nations"[All Fields]) OR (underdeveloped[All Fields] AND ("Nation"[Journal] OR "nation"[All Fields])) OR (underdeveloped[All Fields] AND nations[All Fields]) OR (emergent[All Fields] AND ("Nation"[Journal] OR "nation"[All Fields])) OR (emergent[All Fields] AND nations[All Fields]) OR ("africa"[MeSH Terms] OR "africa"[All Fields]) OR (("poverty"[MeSH Terms] OR "poverty"[All Fields] OR ("low"[All Fields] AND "income"[All Fields]) OR "low income"[All Fields]) AND countries[All Fields]) OR (("poverty"[MeSH Terms] OR "poverty"[All Fields] OR ("low"[All Fields] AND "income"[All Fields]) OR "low income"[All Fields]) AND country[All Fields]) OR (middle[All Fields] AND ("income"[MeSH Terms] OR "income"[All Fields]) AND countries[All Fields]) OR (middle[All Fields] AND ("income"[MeSH Terms] OR "income"[All Fields]) AND country[All Fields]) OR (("poverty"[MeSH Terms] OR "poverty"[All Fields] OR "poor"[All Fields]) AND setting[All Fields]) OR (("poverty"[MeSH Terms] OR "poverty"[All Fields] OR "poor"[All Fields]) AND settings[All Fields]) OR (("health resources"[MeSH Terms] OR ("health"[All Fields] AND "resources"[All Fields]) OR "health resources"[All Fields] OR "resource"[All Fields]) AND limited[All Fields] AND setting[All Fields]) OR (("health resources"[MeSH Terms] OR ("health"[All Fields] AND "resources"[All Fields]) OR "health resources"[All Fields] OR "resource"[All Fields]) AND limited[All Fields] AND settings[All Fields]) OR (("health resources"[MeSH Terms] OR ("health"[All Fields] AND "resources"[All Fields]) OR "health resources"[All Fields] OR "resource"[All Fields]) AND scarce[All Fields] AND setting[All Fields]) OR (("health resources"[MeSH Terms] OR ("health"[All Fields] AND "resources"[All Fields]) OR "health resources"[All Fields] OR "resource"[All Fields]) AND scarce[All Fields] AND settings[All Fields]) OR (resource-limited[All Fields] AND setting[All Fields]) OR (resource-limited[All Fields] AND settings[All Fields]) OR (resource-scarce[All Fields] AND setting[All Fields]) OR (resource-scarce[All Fields] AND settings[All Fields]) OR (("poverty"[MeSH Terms] OR "poverty"[All Fields] OR "poor"[All Fields]) AND country[All Fields]) OR (("poverty"[MeSH Terms] OR "poverty"[All Fields] OR "poor"[All Fields]) AND countries[All Fields]) OR (("poverty"[MeSH Terms] OR "poverty"[All Fields] OR ("low"[All Fields] AND "income"[All Fields]) OR "low income"[All Fields]) AND ("Nation"[Journal] OR "nation"[All Fields])) OR (("poverty"[MeSH Terms] OR "poverty"[All Fields] OR ("low"[All Fields] AND "income"[All Fields]) OR "low income"[All Fields]) AND nations[All Fields]) OR (middle[All Fields] AND ("income"[MeSH Terms] OR "income"[All Fields])

AND ("Nation"[Journal] OR "nation"[All Fields])) OR (middle[All Fields] AND ("income"[MeSH Terms] OR "income"[All Fields]) AND nations[All Fields]) OR (third[All Fields] AND ("WORLD"[Journal] OR "world"[All Fields])) OR ("middle east"[MeSH Terms] OR ("middle"[All Fields] AND "east"[All Fields]) OR "middle east"[All Fields]) OR ("india"[MeSH Terms] OR "india"[All Fields]) OR ("asia"[MeSH Terms] OR "asia"[All Fields]) OR ("europe, eastern"[MeSH Terms] OR ("europe"[All Fields] AND "eastern"[All Fields]) OR "eastern europe"[All Fields] OR ("eastern"[All Fields] AND "europe"[All Fields])) OR ("philippines"[MeSH Terms] OR "philippines"[All Fields]) OR ("taiwan"[MeSH Terms] OR "taiwan"[All Fields]) OR ("indonesia"[MeSH Terms] OR "indonesia"[All Fields]) OR ("latin america"[MeSH Terms] OR ("latin"[All Fields] AND "america"[All Fields]) OR "latin america"[All Fields]) OR ("south america"[MeSH Terms] OR ("south"[All Fields] AND "america"[All Fields]) OR "south america"[All Fields]) OR ("central america"[MeSH Terms] OR ("central"[All Fields] AND "america"[All Fields]) OR "central america"[All Fields]) OR ("china"[MeSH Terms] OR "china"[All Fields]) OR ("russia"[MeSH Terms] OR "russia"[All Fields]))
AND
((Immune[All Fields] AND failure[All Fields]) OR (immunological[All Fields] AND failure[All Fields]) OR (("allergy and immunology"[MeSH Terms] OR ("allergy"[All Fields] AND "immunology"[All Fields]) OR "allergy and immunology"[All Fields] OR "immunologic"[All Fields]) AND failure[All Fields]) OR (immune[All Fields] AND activation[All Fields]) OR (immunological[All Fields] AND activation[All Fields]) OR (("allergy and immunology"[MeSH Terms] OR ("allergy"[All Fields] AND "immunology"[All Fields]) OR "allergy and immunology"[All Fields] OR "immunologic"[All Fields]) AND activation[All Fields]) OR (immune[All Fields] AND modulation[All Fields]) OR (immunological[All Fields] AND modulation[All Fields]) OR (("allergy and immunology"[MeSH Terms] OR ("allergy"[All Fields] AND "immunology"[All Fields]) OR "allergy and immunology"[All Fields] OR "immunologic"[All Fields]) AND modulation[All Fields]) OR ("immune system processes"[MeSH Terms] OR ("immune"[All Fields] AND "system"[All Fields] AND "processes"[All Fields]) OR "immune system processes"[All Fields] OR ("immune"[All Fields] AND "function"[All Fields]) OR "immune function"[All Fields]) OR (immunological[All Fields] AND ("physiology"[Subheading] OR "physiology"[All Fields] OR "function"[All Fields] OR "physiology"[MeSH Terms] OR "function"[All Fields])) OR ("immune system processes"[MeSH Terms] OR ("immune"[All Fields] AND "system"[All Fields] AND "processes"[All Fields]) OR "immune system processes"[All Fields] OR ("immunologic"[All Fields] AND "function"[All Fields]) OR "immunologic function"[All Fields]) OR ("immune system diseases"[MeSH Terms] OR ("immune"[All Fields] AND "system"[All Fields] AND "diseases"[All Fields]) OR "immune system diseases"[All Fields] OR ("immune"[All Fields] AND "dysfunction"[All Fields]) OR "immune dysfunction"[All Fields]) OR (immunological[All Fields] AND ("physiopathology"[Subheading] OR "physiopathology"[All Fields] OR "dysfunction"[All Fields])) OR (("allergy and immunology"[MeSH Terms] OR ("allergy"[All Fields] AND "immunology"[All Fields]) OR "allergy and immunology"[All Fields] OR "immunologic"[All Fields]) AND ("physiopathology"[Subheading] OR "physiopathology"[All Fields] OR "dysfunction"[All Fields])) OR (immune[All Fields] AND restoration[All Fields]) OR (immune[All Fields] AND response[All Fields]) OR (immunological[All Fields] AND response[All Fields]) OR (("allergy and immunology"[MeSH Terms] OR ("allergy"[All Fields] AND "immunology"[All Fields]) OR "allergy and immunology"[All Fields] OR "immunologic"[All Fields]) AND response[All Fields]) OR (immune[All Fields] AND response[All Fields]) OR (immunological[All Fields] AND success[All Fields]) OR (("allergy and immunology"[MeSH Terms] OR ("allergy"[All Fields] AND "immunology"[All Fields]) OR "allergy and immunology"[All Fields] OR "immunologic"[All Fields]) AND success[All Fields]) OR (immune[All Fields] AND outcomes[All Fields]) OR (immunological[All Fields] AND outcomes[All

Fields]) OR (("allergy and immunology"[MeSH Terms] OR ("allergy"[All Fields] AND "immunology"[All Fields]) OR "allergy and immunology"[All Fields] OR "immunologic"[All Fields]) AND outcomes[All Fields]) OR (immune[All Fields] AND recovery[All Fields]) OR (immunological[All Fields] AND recovery[All Fields]) OR (("allergy and immunology"[MeSH Terms] OR ("allergy"[All Fields] AND "immunology"[All Fields]) OR "allergy and immunology"[All Fields] OR "immunologic"[All Fields]) AND recovery[All Fields]) OR ("treatment failure"[MeSH Terms] OR ("treatment"[All Fields] AND "failure"[All Fields]) OR "treatment failure"[All Fields]) OR (("therapeutics"[MeSH Terms] OR "therapeutics"[All Fields] OR "therapeutic"[All Fields]) AND failure[All Fields]) OR ("disease progression"[MeSH Terms] OR ("disease"[All Fields] AND "progression"[All Fields]) OR "disease progression"[All Fields]) OR CD4[All Fields] OR TLC[All Fields] OR ("lymphocyte count"[MeSH Terms] OR ("lymphocyte"[All Fields] AND "count"[All Fields]) OR "lymphocyte count"[All Fields] OR ("total"[All Fields] AND "lymphocyte"[All Fields] AND "count"[All Fields]) OR "total lymphocyte count"[All Fields])))

AND

(Antiretroviral[All Fields] OR ("antiretroviral therapy, highly active"[MeSH Terms] OR ("antiretroviral"[All Fields] AND "therapy"[All Fields] AND "highly"[All Fields] AND "active"[All Fields]) OR "highly active antiretroviral therapy"[All Fields] OR "haart"[All Fields]) OR ("antiretroviral therapy, highly active"[MeSH Terms] OR ("antiretroviral"[All Fields] AND "therapy"[All Fields] AND "highly"[All Fields] AND "active"[All Fields]) OR "highly active antiretroviral therapy"[All Fields] OR ("highly"[All Fields] AND "active"[All Fields] AND "antiretroviral"[All Fields] AND "therapy"[All Fields])) OR (antiretrovirus[All Fields] AND agent[All Fields]) OR (anti[All Fields] AND ("retroviridae"[MeSH Terms] OR "retroviridae"[All Fields] OR "retrovirus"[All Fields]) AND agent[All Fields])))

AND

(("malnutrition"[MeSH Terms] OR "malnutrition"[All Fields]) OR ("malnutrition"[MeSH Terms] OR "malnutrition"[All Fields] OR "malnourished"[All Fields]) OR ("malnutrition"[MeSH Terms] OR "malnutrition"[All Fields] OR "undernutrition"[All Fields]) OR ("malnutrition"[MeSH Terms] OR "malnutrition"[All Fields] OR "undernourished"[All Fields]) OR wasting[All Fields] OR ("starvation"[MeSH Terms] OR "starvation"[All Fields]) OR ("nutritional status"[MeSH Terms] OR ("nutritional"[All Fields] AND "status"[All Fields]) OR "nutritional status"[All Fields]) OR (nutritional[All Fields] AND impairment[All Fields]) OR (nutritional[All Fields] AND modulation[All Fields]) OR ("nutrition assessment"[MeSH Terms] OR ("nutrition"[All Fields] AND "assessment"[All Fields]) OR "nutrition assessment"[All Fields]) OR (nutritional[All Fields] AND parameter[All Fields]) OR nutritional[All Fields] OR ("nutritional status"[MeSH Terms] OR ("nutritional"[All Fields] AND "status"[All Fields]) OR "nutritional status"[All Fields] OR "nutrition"[All Fields] OR "nutritional sciences"[MeSH Terms] OR ("nutritional"[All Fields] AND "sciences"[All Fields]) OR "nutritional sciences"[All Fields]) OR ("body mass index"[MeSH Terms] OR ("body"[All Fields] AND "mass"[All Fields] AND "index"[All Fields]) OR "body mass index"[All Fields]) OR BMI[All Fields] OR anthropometric[All Fields] OR (mid-upper[All Fields] AND ("arm"[MeSH Terms] OR "arm"[All Fields])) OR MUAC[All Fields] OR (midarm[All Fields] AND ("muscles"[MeSH Terms] OR "muscles"[All Fields] OR "muscle"[All Fields])) OR (("skin"[MeSH Terms] OR "skin"[All Fields]) AND fold[All Fields]) OR skinfold[All Fields] OR (("human body"[MeSH Terms] OR ("human"[All Fields] AND "body"[All Fields]) OR "human body"[All Fields] OR "body"[All Fields]) AND ("cells"[MeSH Terms] OR "cells"[All Fields] OR "cell"[All Fields]) AND ("molecular weight"[MeSH Terms] OR ("molecular"[All Fields] AND "weight"[All Fields]) OR "molecular weight"[All Fields] OR "mass"[All Fields])) OR BCM[All Fields] OR "intracellular water"[All Fields] OR ("Intensive Care World"[Journal] OR "icw"[All Fields]) OR "extracellular water"[All Fields] OR ECW[All Fields] OR ("fat body"[MeSH Terms] OR ("fat"[All

Fields] AND "body"[All Fields]) OR "fat body"[All Fields] OR ("body"[All Fields] AND "fat"[All Fields]) OR "body fat"[All Fields] OR "adipose tissue"[MeSH Terms] OR ("adipose"[All Fields] AND "tissue"[All Fields]) OR "adipose tissue"[All Fields] OR ("body"[All Fields] AND "fat"[All Fields])) OR (non-fat[All Fields] AND ("molecular weight"[MeSH Terms] OR ("molecular"[All Fields] AND "weight"[All Fields]) OR "molecular weight"[All Fields] OR "mass"[All Fields])) OR (fat-free[All Fields] AND ("molecular weight"[MeSH Terms] OR ("molecular"[All Fields] AND "weight"[All Fields]) OR "molecular weight"[All Fields] OR "mass"[All Fields])) OR FFM[All Fields] OR (lean[All Fields] AND ("human body"[MeSH Terms] OR ("human"[All Fields] AND "body"[All Fields]) OR "human body"[All Fields] OR "body"[All Fields]) AND ("molecular weight"[MeSH Terms] OR ("molecular"[All Fields] AND "weight"[All Fields]) OR "molecular weight"[All Fields] OR "mass"[All Fields])) OR (fat[All Fields] AND ("molecular weight"[MeSH Terms] OR ("molecular"[All Fields] AND "weight"[All Fields]) OR "molecular weight"[All Fields] OR "mass"[All Fields])) OR ("Field methods"[Journal] OR "fm"[All Fields]) OR (total[All Fields] AND ("body water"[MeSH Terms] OR ("body"[All Fields] AND "water"[All Fields]) OR "body water"[All Fields])) OR ("electric impedance"[MeSH Terms] OR ("electric"[All Fields] AND "impedance"[All Fields]) OR "electric impedance"[All Fields] OR ("bioelectrical"[All Fields] AND "impedance"[All Fields]) OR "bioelectrical impedance"[All Fields]) OR BIA[All Fields] OR bioimpedance[All Fields] OR ("anaemia"[All Fields] OR "anemia"[MeSH Terms] OR "anemia"[All Fields]) OR ("haemoglobin"[All Fields] OR "hemoglobins"[MeSH Terms] OR "hemoglobins"[All Fields] OR "hemoglobin"[All Fields]) OR CRP[All Fields] OR ("c-reactive protein"[MeSH Terms] OR ("c-reactive"[All Fields] AND "protein"[All Fields]) OR "c-reactive protein"[All Fields] OR "c reactive protein"[All Fields]) OR ("weight loss"[MeSH Terms] OR ("weight"[All Fields] AND "loss"[All Fields]) OR "weight loss"[All Fields]) OR ("weight gain"[MeSH Terms] OR ("weight"[All Fields] AND "gain"[All Fields]) OR "weight gain"[All Fields]) OR ("albumins"[MeSH Terms] OR "albumins"[All Fields] OR "albumin"[All Fields]) OR ("hypoalbuminaemia"[All Fields] OR "hypoalbuminemia"[MeSH Terms] OR "hypoalbuminemia"[All Fields]))

AND

("1980/01/01"[PDAT]: "2013/12/31"[PDAT])

2. EMBASE (OVID interface)

The “explode” option to the Emtree terms - MeSH equivalent – was not applied to EMBASE research due to excessive noise.

Database: Embase <1980 to 2013 June 06>
Search Strategy:
----
1 malnutrition*.af. (54802)
2 malnourished.af. (9321)
3 undernutrition*.af. (6051)
4 undernourished.af. (3013)
5 wasting.af. (15886)
6 starvation*.af. (28837)
7 nutritional status.af. (46676)
8 nutritional impairment.af. (111)
9 nutritional modulation.af. (193)

10 nutrition* assessment*.af. (16613)
11 nutritional parameter*.af. (2521)
12 nutrition*.ti. (89743)
13 nutrition*.sh. (184849)
14 nutrition*.ab. (180560)
15 body mass index.af. (126120)
16 BMI.af. (121646)
17 anthropometric*.af. (38322)
18 mid-upper arm.af. (869)
19 MUAC.af. (405)
20 midarm muscle.af. (195)
21 skin fold.af. (1821)
22 skinfold.af. (14170)
23 body cell mass.af. (860)
24 BCM.af. (1721)
25 intracellular water.af. (1156)
26 ICW.af. (664)
27 extracellular water.af. (1323)
28 ECW.af. (682)
29 non-fat mass.af. (10)
30 fat-free mass.af. (6078)
31 lean body mass.af. (6845)
32 fat mass.af. (15795)
33 total body water.af. (3753)
34 bioelectrical impedance.af. (3800)
35 BIA.af. (3467)
36 bioimpedance*.af. (2986)
37 albumin.af. (183506)
38 hypoalbuminemia.af. (8433)
39 anemia.af. (235938)
40 hemoglobin.af. (219344)
41 c reactive protein.af. (86197)
42 CRP.af. (42717)
43 weight loss.af. (73314)
44 weight gain.af. (82812)
45 or/1-44 (1302626)
46 antiretroviral*.af. (58438)
47 HAART.af. (13866)
48 highly active antiretroviral therapy.af. (31493)
49 antiretrovirus agent.af. (32690)
50 or/46-49 (68802)
51 immun* failure*.af. (486)
52 immun* activation*.af. (8805)
53 immun* modulation*.af. (3773)
54 immun* function*.af. (30402)
55 immun* dysfunction*.af. (4063)
56 immun* restoration*.af. (698)
57 immun* response*.af. (273581)

58 immun* success.af. (80)
59 immun* outcome*.af. (731)
60 immun* recover*.af. (1474)
61 treatment* failure*.af. (89850)
62 therapeutic failure*.af. (3206)
63 disease* progression*.af. (57814)
64 CD4.af. (176117)
65 TLC.af. (18010)
66 total lymphocyte count.af. (911)
67 or/51-66 (600219)
68 developing countr*.af. (101771)
69 underdeveloped countr*.af. (924)
70 emergent countr*.af. (21)
71 developing nation*.af. (2245)
72 underdeveloped nation*.af. (67)
73 emergent nation*.af. (6)
74 africa.af. (184394)
75 low income countr*.af. (3075)
76 poor setting*.af. (1892)
77 middle income countr*.af. (3871)
78 resource limited setting*.af. (2635)
79 resource scarce setting*.af. (30)
80 resource-scarce setting*.af. (30)
81 poor countr*.af. (1938)
82 low income nation*.af. (47)
83 middle income nation*.af. (39)
84 third world.af. (3274)
85 Middle East.af. (13028)
86 India.af. (540142)
87 asia.af. (92513)
88 Eastern Europe.af. (7634)
89 Philippines.af. (15374)
90 Taiwan.af. (189681)
91 Indonesia.af. (17214)
92 Latin America.af. (10263)
93 South America.af. (16418)
94 Central America.af. (18874)
95 china.af. (1061618)
96 russia.af. (175040)
97 or/68-96 (2143170)
98 and/45,50,67,97 (940)
99 limit 98 to embase (851)

****

3. Cochrane Central (OVID interface)

Database: EBM Reviews - Cochrane Database of Systematic Reviews <2005 to September 2013>, EBM Reviews - ACP Journal Club <1991 to September 2013>, EBM Reviews - Database of Abstracts of Reviews of Effects <3rd Quarter 2013>, EBM Reviews - Cochrane Central Register of Controlled Trials <September 2013>, EBM Reviews - Cochrane Methodology Register <3rd Quarter 2012>, EBM Reviews - Health Technology Assessment <3rd Quarter 2013>, EBM Reviews - NHS Economic Evaluation Database <3rd Quarter 2013>

We specifically hand-searched relevant abstracts in the following review groups : Cochrane HIV Group and Cochrane Public Health Group.

Search Strategy:

----

1 malnutrition*.af. (1436)
2 malnourished.af. (717)
3 undernutrition*.af. (163)
4 undernourished.af. (199)
5 wasting.af. (485)
6 starvation*.af. (182)
7 nutritional status.af. (2561)
8 nutritional impairment.af. (3)
9 nutritional modulation.af. (11)
10 nutrition* assessment*.af. (510)
11 nutritional parameter*.af. (185)
12 nutrition*.ti. (4513)
13 nutrition*.sh. (3093)
14 nutrition*.ab. (8042)
15 body mass index.af. (11399)
16 BMI.af. (5741)
17 anthropometric*.af. (1883)
18 mid-upper arm.af. (92)
19 MUAC.af. (30)
20 midarm muscle.af. (21)
21 skin fold.af. (161)
22 skinfold.af. (698)
23 body cell mass.af. (81)
24 BCM.af. (62)
25 intracellular water.af. (46)
26 ICW.af. (19)
27 extracellular water.af. (96)
28 ECW.af. (44)
29 non-fat mass.af. (1)
30 fat-free mass.af. (695)
31 lean body mass.af. (858)

32 fat mass.af. (1237)
33 total body water.af. (209)
34 bioelectrical impedance.af. (211)
35 BIA.af. (138)
36 bioimpedance*.af. (195)
37 albumin.af. (5505)
38 hypoalbuminemia.af. (107)
39 anemia.af. (5628)
40 hemoglobin.af. (9003)
41 c reactive protein.af. (4896)
42 CRP.af. (2362)
43 weight loss.af. (6545)
44 weight gain.af. (4848)
45 or/1-44 (51813)
46 antiretroviral*.af. (3283)
47 HAART.af. (627)
48 highly active antiretroviral therapy.af. (653)
49 antiretrovirus agent.af. (87)
50 or/46-49 (3357)
51 immun* failure*.af. (26)
52 immun* activation*.af. (178)
53 immun* modulation*.af. (93)
54 immun* function*.af. (1378)
55 immun* dysfunction*.af. (82)
56 immun* restoration*.af. (17)
57 immun* response*.af. (3763)
58 immun* success.af. (8)
59 immun* outcome*.af. (121)
60 immun* recover*.af. (70)
61 treatment* failure*.af. (6259)
62 therapeutic failure*.af. (301)
63 disease* progression*.af. (7190)
64 CD4.af. (4724)
65 TLC.af. (297)
66 total lymphocyte count.af. (86)
67 or/51-66 (21997)
68 developing countr*.af. (2788)
69 underdeveloped countr*.af. (26)
70 emergent countr*.af. (0)
71 developing nation*.af. (85)
72 underdeveloped nation*.af. (2)
73 emergent nation*.af. (0)
74 africa.af. (4242)
75 low income countr*.af. (435)
76 poor setting*.af. (177)
77 middle income countr*.af. (553)
78 resource limited setting*.af. (172)
79 resource scarce setting*.af. (3)

80 resource-scarce setting*.af. (3)
81 poor countr*.af. (104)
82 low income nation*.af. (5)
83 middle income nation*.af. (6)
84 third world.af. (92)
85 Middle East.af. (232)
86 India.af. (6491)
87 asia.af. (1725)
88 Eastern Europe.af. (106)
89 Philippines.af. (407)
90 Taiwan.af. (3629)
91 Indonesia.af. (557)
92 Latin America.af. (427)
93 South America.af. (418)
94 Central America.af. (94)
95 china.af. (11723)
96 russia.af. (782)
97 or/68-96 (28896)
98 and/45,50,67,97 (70)

****
