# Supplementary material for: Association between nutritional status and the immune response in HIV + patients under HAART: protocol for a systematic review
Source: Syst Rev. 2014 Feb 10;3:9. doi: 10.1186/2046-4053-3-9 (PMC3922999; doi:10.1186/2046-4053-3-9)
Supplement: Additional file 2 — Endnote file manipulations. [file 2046-4053-3-9-S2.pdf]

Annexe 2. Endnote file manipulations

.

1. Entries uncovered by search strategies will be combined in an Endnote file
2. Duplicates will be removed
3. Entries from before 1996 will be removed (*year the use of highly active antiretroviral therapy (HAART) consisting of three antiretroviral molecules became standard)*
4. Removal of peri/postpartum literature
    a. Endnote search strategies (match words)
        i. *Pregnant (title) OR Maternal (title) OR Pregnancy (title) OR breastfeeding (title) OR postpartum (title) OR mother (title) OR mothers(title) OR postnatal (title)*
    b. Manual revision of all identified entries before deletion to insure that no pertinent entries would be removed in the process. Entries not strictly studying peri/postpartum issues were returned to the main endnote file.
5. Removal of pediatrics literature
    a. Endnote search strategies (match words)
        i. *Child (title) OR Children (title) OR Neonate (title) OR pediatric (title) OR vertical (title) OR infant (title) OR infants (title) OR paediatric (title) NOT adults (any field)*
    b. Manual revision of all identified entries before deletion to insure that no pertinent entries would be removed in the process. Entries not strictly studying pediatric issues were returned to the main endnote file.
