# Supplementary material for: Association between nutritional status and the immune response in HIV + patients under HAART: protocol for a systematic review
Source: Syst Rev. 2014 Feb 10;3:9. doi: 10.1186/2046-4053-3-9 (PMC3922999; doi:10.1186/2046-4053-3-9)
Supplement: Additional file S — Data collection form. [file 2046-4053-3-9-S3.pdf]

Data collection form

Notes:

- Be consistent in the order and style used to describe each report.
- Record any missing or unclear information so to indicate that the information was not found in the study report(s) but not forgotten.

Review title	Association between nutritional status and the immune response in HIV+ patients under HAART: Protocol for a systematic review
Study ID *(surname of first author and year first full report of study was published e.g. Smith 2001)*	
Notes	

General Information

Date form completed *(dd/mm/yyyy)*	
Name of person extracting data	
Reference citation (e.g. Medline)	
Study author contact details	
Type of publication<br>*(e.g. full report, abstract, letter)*	
Notes:	

Study eligibility

Study Characteristics	Eligibility criteria	Eligibility criteria met? Yes	No	Unclear/ unspecified	Location in text or source *(pg & ¶/fig/table)*
Type of study	Experimental study including randomized controlled trials (RCTs) or cluster-randomized trials (CRTs).	☐	☐	☐	
	Quasi-experimental studies including quasi-randomized trials, controlled before-after studies (CBAs) and interrupted time series studies (ITSs).	☐	☐	☐	
	Observational studies including cohort, case-control and cross-sectional studies.	☐	☐	☐	
Participants and setting	HIV-1 infectedadults	☐	☐	☐	
	Non pregnant	☐	☐	☐	
	Low or middle income country setting (as listed in the *World Bank Group's classification of countries by income groups (see Annexe))*	☐	☐	☐	
	Naive to ART (if initiating treatment at T0) or under ART < 1 year	☐	☐	☐	
Types of comparison	Undernourished vs well nourished *(according to a predefined cutoff value)*	☐	☐	☐	
Types of outcome measures	**Primary outcome:**				
	1) CD4 counts or CD4%	☐	☐	☐	
	2) *Change in CD4 counts between timepoints*	☐	☐	☐	
	**Secondary outcomes:**				
	1) Immune failure	☐	☐	☐	
	2) Time to immune failure	☐	☐	☐	
Types of determinants	**Body composition**	☐	☐	☐	
	1) Weight	☐	☐	☐	
	2) BMI	☐	☐	☐	
	3) MUAC	☐	☐	☐	
	4) Skinfold	☐	☐	☐	
	5) BIA (bioelectrical impedance)	☐	☐	☐	
	**Inflammation**				
	1) Albumin	☐	☐	☐	
	2) Hemoglobin	☐	☐	☐	

	3) CRP	☐ ☐ ☐	
Results	Associative measure between one or more dichotomized/categorized nutritional markers OR weight change AND immune response	☐ ☐ ☐	
INCLUDE ☐	EXCLUDE ☐	UNCERTAIN ☐	
Reason for exclusion			
Notes:			

DO NOT PROCEED IF STUDY EXCLUDED FROM REVIEW

Characteristics of included studies

Methods

	**Descriptions as stated in report/paper**	**Location in text or source** *(pg & ¶/fig/table)*
**Objectives**		
**Design**		
**Unit of observation**		
**Start date**		
**End date**		

**Duration of participation**<br><br>*(from recruitment to last follow-up)*		
**Ethical approval needed/ obtained for study**	☐ Yes ☐ No ☐ Unclear	
**Notes:**		

Participants and immune response

	Description	Location in text or source *(pg & ¶/fig/table)*
Population description *(from which study participants were drawn)*		
Setting and context<br><br>*(For example, as part of a RCT of nutritional intervention, ...)*		
Inclusion criterions		
Exclusion criterions		
Method of recruitment of participants *(e.g. phone, mail, clinic patients)*		
Informed consent obtained	☐ Yes ☐ No ☐ Unclear/ not indicated	

Total no. of subjects	Included:______________________<br><br>Excluded:______________________	
Participation agreement (%)		
Clusters<br>*(if applicable, no., type, no. people per cluster)*		
Baseline discrepancies<br>*(if applicable)*		
Lost to follow-up	How many:_____________________<br><br>Characteristics of those LTFU:<br>____<br>____<br><br>Statistically different from those who remained?<br><br>Yes ☐ No ☐ Not indicated ☐	
Mortality	How many:_____________________<br><br>Characteristics of those LTFU:<br>____<br>____<br><br>Statistically different from those who remained?<br><br>Yes ☐ No ☐ Not indicated ☐	
Withdrawals	How many:_____________________<br><br>Characteristics of those LTFU:<br>____<br>____<br><br>Statistically different from those who remained?<br><br>Yes ☐ No ☐ Not indicated ☐	
Number of total person-years (if applicable)		
Missing data		

<table>
<tr><td rowspan="2">Outcome(s)

Definition, measure & classification</td><td><u>Primary outcome</u>
1) Absolute CD4 counts
2) Relative CD4 counts (% change in CD4 between timepoints)

Indicate if used as continuous or categorized (indicate cut-off points used by the authors):
____
____
____

Method used to measure CD4 at each time points:
1) FACS counter ☐
2) Other
____
____</td><td></td></tr>
<tr><td><u>Secondary outcomes</u>
1) Immune failure
2) Time to immune failure

Operational definition of immune failure used by the authors:
____
____
____
____</td><td></td></tr>
<tr><td rowspan="2">Determinants</td><td>Body composition</td><td rowspan="2"></td></tr>
<tr><td>Weight
<u>Methods of assessment</u>
1. Was the weight auto-reported or measured by an expert
Auto-reported ☐ Uncertain ☐
Measured by expert ☐ Not indicated ☐

Comments:_________________________________</td></tr>
</table>

	**BMI**<br>*Indicate cut-off points used to categorized this variable*<br>____<br>____<br>____<br><br><u>Methods of assessment</u><br>*1. Did the observer receive a formal training in BMI measurement*<br>Yes ☐ Uncertain ☐<br>No ☐ Not indicated ☐<br><br>*Comments:*________________________________<br><br>2. If longitudinal measurements: Did the same observer take all measurements for a same patient<br>Yes ☐ Uncertain ☐<br>No ☐ Not indicated ☐<br>*Comments:*________________________________	
	**MUAC** (mid upper arm circumference)<br>*Indicate cut-off points used to categorized this variable*<br>____<br>____<br>____<br><br><u>Methods of assessment</u><br>*1. Did the observer receive a formal training in MUAC measurement*<br>Yes ☐ Uncertain ☐<br>No ☐ Not indicated ☐<br>*Comments:*________________________________<br><br>2. If longitudinal measurements: Did the same observer take all measurements for a same patient<br>Yes ☐ Uncertain ☐<br>No ☐ Not indicated ☐<br>*Comments:*________________________________<br><br>*3. At each timepoint, was the measurement repeated 2-3 times and mean value measured*<br>Yes ☐ Uncertain ☐<br>No ☐ Not indicated ☐<br>*Comments:*________________________________	

	**Skinfold**<br>*Indicate cut-off values used to categorized this variable*<br>____<br>____<br>____<br><br><u>Methods of assessment</u><br>1. Specify anatomical location(s): ____________________<br>____<br><br>*2.* Which brand of Calliper were used<br>Brand: ________________________________<br>Not specified ☐<br><br>*3.* Did the observer receive a formal training in SK measurement<br>Yes ☐ Uncertain ☐<br>No ☐ Not indicated ☐<br>*Comments:* ___________________________________<br><br>*3.* Was the measurement technique calibrated by a second expert in the course of the study<br>Yes ☐ Uncertain ☐<br>No ☐ Not indicated ☐<br>*Comments:* ___________________________________<br><br>4. If longitudinal measurements: Did the same observer take all measurements for a same patient<br>Yes ☐ Uncertain ☐<br>No ☐ Not indicated ☐<br>*Comments:*___________________________________<br><br>*5.* At each time point, was the measurement repeated 2-3 times and mean value measured<br>Yes ☐ Uncertain ☐<br>No ☐ Not indicated ☐<br>*Comments:*___________________________________	

<table>
<tr><td></td><td>BIA/BIS (bioelectrical impedance)
<u>Indicate which compartments were measured/estimated using this technique</u>
• BCM ☐
• TBW ☐
• FFM ☐
• ICW ☐
• ECW ☐
• Phase angle
<u>Methods of assessment</u>
1. Were the following factors evaluated and/or taken into account
• Consumption of water Yes ☐ No ☐
• Consumption of food Yes ☐ No ☐
• Physical activity in hours before measure Yes ☐ No ☐
• Ambient temperature Yes ☐ No ☐
• Medical conditions affecting electrolytes Yes ☐ No ☐

2. What was the position of the electrodes
• Hand-to-foot ☐
• Hand-to-hand ☐
• Foot-to-foot ☐
• Not specified ☐

3. Which BIA/BIS equation was used
____
____
____
Not specified ☐

4. If applicable, is the predictive equation used validated for the study population
Yes, specified by the authors ☐
No, specified by the authors ☐
Not specified ☐</td><td></td></tr>
<tr><td></td><td>Inflammatory markers</td><td></td></tr>
<tr><td></td><td>Albumin
Indicate cut-off values used to categorized this variable
____
____
____

<u>Methods of assessment</u>
1. Was the serum sample
Fresh ☐
Frozen ☐
Not indicated ☐

2. Technique used to measure level of albumin
____
____</td><td></td></tr>
</table>

<table>
<tr><td rowspan="2"></td><td>Haemoglobin
Indicate cut-off values used to categorized this variable
____
____
____

<u>Methods of assessment</u>
1. Source of blood
Capillary ☐
Venous ☐
Not indicated ☐
2. Assessment method
HemoCue ☐
Cyanmethemoglobin ☐
Other ☐
Specify:_______________
Not indicated ☐</td><td></td></tr>
<tr><td>CRP
Indicate cut-off values used to categorized this variable
____
____
____

<u>Methods of assessment</u>
1. Type of test
Low Sensitivity ☐
High Sensitivity ☐
Not indicated ☐</td><td></td></tr>
<tr><td>Confounding factors/ effect modifiers accounted for in the analyses</td><td></td><td></td></tr>
<tr><td rowspan="2">Results
(specify, e.g. OR, RR, IRR)
(specify the reference group)</td><td>Crude</td><td></td></tr>
<tr><td>Adjusted</td><td></td></tr>
<tr><td>Reported limitations of study's methods/results</td><td></td><td></td></tr>
</table>

**Key conclusions** *(as stated in report/paper)*		
**Scientific quality** (specify tool, e.g. modified EPHPP tool)<br><br>authors		
Notes:		

Other information

Study funding sources *(including role of funders)*		
Possible conflicts of interest *(for study authors)*		
References of relevant studies		
Correspondence required for further study information *(from whom, what and when)*		
Notes:		

Appendix

The World Bank Group's classification of countries by income groups

Available from:

http://data.worldbank.org/about/country-classifications/country-and-lending-groups

Low-income economies

Afghanistan	Gambia, The	Mozambique
Bangladesh	Guinea	Myanmar
Benin	Guinea-Bisau	Nepal
Burkina Faso	Haiti	Niger
Burundi	Kenya	Rwanda
Cambodia	Korea, Dem Rep.	Sierra Leone
Central African Republic	Kyrgyz Republic	Somalia
Chad	Liberia	Tajikistan
Comoros	Madagascar	Tanzania
Congo, Dem. Rep	Malawi	Togo
Eritrea	Mali	Uganda
Ethiopia	Mauritania	Zimbabwe

Lower-middle-income economies

Albania	Indonesia	Samoa
Armenia	India	São Tomé and Principe
Belize	Iraq	Senegal
Bhutan	Kiribati	Solomon Islands
Bolivia	Kosovo	South Sudan
Cameroon	Lao PDR	Sri Lanka
Cape Verde	Lesotho	Sudan
Congo, Rep.	Marshall Islands	Swaziland
Côte d'Ivoire	Micronesia, Fed. Sts.	Syrian Arab Republic
Djibouti	Moldova	Timor-Leste
Egypt, Arab Rep.	Mongolia	Tonga
El Salvador	Morocco	Ukraine
Fiji	Nicaragua	Uzbekistan
Georgia	Nigeria	Vanuatu
Ghana	Pakistan	Vietnam
Guatemala	Papua New Guinea	West Bank and Gaza
Guyana	Paraguay	Yemen, Rep.
Honduras	Philippines	Zambia

Upper-middle-income economies

Angola
Algeria
American Samoa
Antigua and Barbuda
Argentina
Azerbaijan
Belarus
Bosnia and Herzegovina
Botswana
Brazil
Bulgaria
Chile
China
Colombia
Costa Rica
Cuba
Dominica
Dominican Republic
Ecuador
Gabon
Grenada
Iran, Islamic Rep.
Jamaica
Jordan
Kazakhstan
Latvia
Lebanon
Libya
Lithuania
Macedonia, FYR
Malaysia
Maldives
Mauritius
Mexico
Montenegro
Namibia
Palau
Panama
Peru
Romania
Russian Federation
Serbia
Seychelles
South Africa
St. Lucia
St. Vincent and the Grenadines
Suriname
Thailand
Tunisia
Turkey
Turkmenistan
Tuvalu
Uruguay
Venezuela, RB
